# Phytochemical Targeting of STAT3 Orchestrated Lipid Metabolism in Therapy-Resistant Cancers

**DOI:** 10.3390/biom10081118

**Published:** 2020-07-28

**Authors:** Carmen Tse, Ashleigh Warner, Rufaik Farook, James G Cronin

**Affiliations:** Immunometabolism & Cancer Research Group, Institute of Life Science, Swansea University Medical School, Singleton Park Campus, Swansea SA2 8PP, Wales, UK; 793815@swansea.ac.uk (C.T.); 920584@swansea.ac.uk (A.W.); 688317@swansea.ac.uk (R.F.)

**Keywords:** STAT3, lipids, cancer, phytochemical

## Abstract

Lipids are critical for maintaining homeostasis and cellular metabolism. However, the dysregulation of lipid metabolism contributes to the pathogenesis of chronic inflammatory diseases and is a hallmark of several cancer types. Tumours exist in a microenvironment of poor vascularization-depleted oxygen and restricted nutrients. Under these conditions, tumours have been shown to increasingly depend on the metabolism of fatty acids for sustained proliferation and survival. Signal transducer and activator of transcription 3 (STAT3) plays a key role in cellular processes such as cell growth, apoptosis and lipid metabolism. Aberrant STAT3 activity, as seen in several cancer types, is associated with tumour progression and malignancy, in addition to propagating crosstalk between tumour cells and the microenvironment. Furthermore, STAT3-regulated lipid metabolism is critical for cancer stem cell self-renewal and therapy resistance. Plant-derived compounds known as phytochemicals are a potential source for novel cancer therapeutic drugs. Dietary phytochemicals are known to modulate key cellular signalling pathways involved in lipid homeostasis and metabolism, including the STAT3 signalling pathways. Targeting STAT3 orchestrated lipid metabolism has shown therapeutic promise in human cancer models. In this review, we summarize the antitumour activity of phytochemicals with an emphasis placed on their effect on STAT3-regulated lipid metabolism and their role in abrogating therapy resistance.

## 1. Introduction

The metabolic landscape of tumours is complex and cancer cells must adapt their metabolism to meet the biosynthetic and energetic demands that are required to sustain rapid growth, initiate metastasis and evade antitumour immunity. To fulfil this demand, cancer cells demonstrate metabolic flexibility, adapting to the often-harsh tumour microenvironment. For instance, cancer cells display the ability to take up lipids from the extracellular environment, undergo lipolysis, as well as synthesize lipids de novo [1]. Studies have shown that the Janus kinase (JAK)—signal transducer and activator of transcription 3 (STAT3) pathway—plays a critical role in lipid metabolism, cytokine production, and inflammation driving tumourigenesis and metastasis [2,3,4,5]. In many types of cancers STAT3 is constitutively activated and STAT3 inhibitors have shown promise as antitumour therapies, however, few have entered clinical trials due to a lack of efficacy as a result of adverse side effects and toxicity [6]. Consequently, alternative or complimentary therapies targeting STAT3 signalling in metabolically flexible cancers would be beneficial.

**Figure 1:**
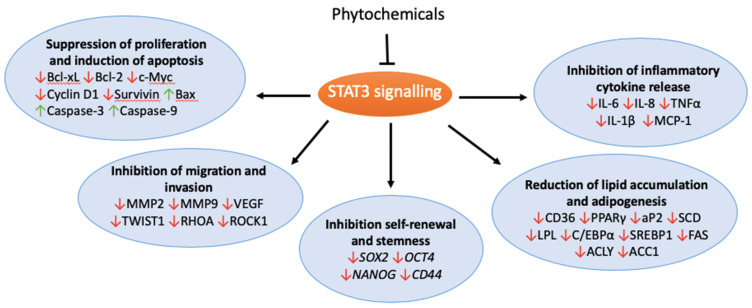
STAT3 signalling in tumourigenesis.

The consumption of fruit and vegetables has been associated with a reduced risk of various types of cancers [7]. Phytochemicals, and plant-derived bioactive compounds found in abundance in fruits and vegetables, may contribute to cancer prevention by regulating processes including metastasis, cytokine production, inflammation and lipid metabolism [8,9]. Disrupting lipid regulation using natural anti-obesity agents has shown promise in targeting cancers [10,11,12]. Phytochemicals show promise through the mediation of various STAT3 signalling pathways important in tumourigenesis (Figure 1). This review will discuss the role of the phytochemical targeting of STAT3 signalling-orchestrated lipid metabolism in improving therapeutic outcomes in cancer.

### 1.1. Lipid Metabolism in Cancer

Metabolic flexibility enables tumour cells to generate adenosine triphosphate (ATP), the cell’s main energy-providing molecule, whilst also committing resources to the cellular pathways that provide the essential building-blocks for cancer cell survival, growth and proliferation. Tumours consist of heterogenous populations of cancer cells, and importantly, a growing body of evidence indicates that metabolic cooperation in the tumour microenvironment between cells with different but complimentary metabolic profiles drives tumour progression [13,14]. Cells mainly use glucose to generate ATP; glucose is metabolized in cells via glycolysis, generating lactate in the cytoplasm, or in aerobic cells through the further metabolism of pyruvate via the tricarboxylic (TCA) cycle and oxidative phosphorylation (OXPHOS) in the mitochondria. Cancer cells frequently use both pathways to generate energy, however, depending on nutrient and oxygen availability, cancer cells have the ability to adapt metabolically. For instance, glucose or glutamine metabolism also provide acetyl CoA for the biosynthesis of fatty acids (FAs) via a truncated TCA cycle in tumour cells, diverting citrate to the cytosol for the production of acetyl-CoA for fatty acid synthesis. Lipids can also be transferred from adipose tissue to be metabolized by neighbouring cancer cells for fatty acid oxidation (FAO; β-oxidation) [14,15,16,17,18]. This metabolic flexibility enables cells within differing nutrient and oxygen niches to become metabolically coupled, promoting cancer cell proliferation and tumour growth.

Lipids circulate in the bloodstream complexed with proteins or FAs. Mammals produce select FAs, whereas other essential FAs require uptake, including polyunsaturated fatty acids (PUFAs), or are synthesized by commensal bacteria in the gut, such as short-chain FAs [19]. FAs are a group of compounds comprised of monoacylglycerols, diacylglycerols and triacylglycerols that are formed from the repeated condensation and reduction of acetyl-CoA upon addition to malonyl-CoA [20]. Triacylglycerols in particular are important to cancer cells as an energy source that can be rapidly exploited to generate ATP via FAO. Lipoprotein lipase (LPL) catalyses the hydrolysis of triacylglycerols into free FAs. FAO is often favoured by cancer cells over other metabolic pathways, even under nutrient replete conditions [21,22]. A variety of plant extracts have been demonstrated to reduce triacylglycerol levels in serum and will be discussed in more detail later in the Review [23,24,25,26,27,28].

In addition to providing cancer cells with energy, lipids are the integral components of biological membranes and cell signalling pathways. Lipid mediators, such as prostaglandins derived from essential FAs, play roles in immunosuppression and tumour progression [29]. For example, arachidonic acid (AA), a long-chain PUFA and a major component of animal fats, is predominantly taken up by the fatty acid translocase CD36, mediating inflammation and acting as a precursor for the synthesis of biologically active prostaglandins [30,31,32]. Prostaglandins and leukotrienes are pro-inflammatory lipids that stimulate tumour-surrounding epithelial cells and stromal cells to produce growth factors promoting a switch to a ‘tumour-supporting’ microenvironment [31].

Arachidonic acid has long been associated with the development and recurrence of various cancers and lipidomic analysis has identified AA as a chemo-protective mediator [32,33]. The quantitative analysis of AA in a spheroid colorectal cancer model revealed that the levels of AA were two-fold higher at the surface of the spheroid than in the medial region, initiating speculation that AA may be contributing to cancer cell migration [34]. This is supported by the result that Berberine, an AA metabolic pathway inhibitor, reduced the volume and weight of tumours in a transplanted mouse model of liver cancer and reduced ovarian cancer cell migration in an in vitro Transwell system model [35,36]. Furthermore, increasing concentrations of AA significantly increase the secretion of the adipokines interleukin 6 (IL-6) and monocyte chemotactic protein (MCP-1) from adipocytes in vitro [37].

### 1.2. Adipokines

As discussed previously, the cancer cells from several types of cancers show metabolic cooperation with adipocytes, including leukemia, melanoma, breast, colorectal and ovarian cancers [14,15,16,17,18]. Furthermore, the coculture of cancer cells with adipocytes results in FAO and an increased secretion of pro-inflammatory adipokines, such as IL-6, MCP-1 and IL-8 [14,15,17]. Blocking the IL-6 receptor (IL-6R) or IL-8 receptor (CXCR1) reduces the adhesion of ovarian cancer cells to the omentum, a fat-rich tissue primarily composed of adipocytes, and reduces the migration of ovarian cancer cells to primary adipocytes in vitro, indicating that the metabolic cooperation between cancer cells and adipocytes is mediated by adipokine signalling molecules [14].

### 1.3. IL-6R/JAK/STAT3 Signalling

**Figure 2:**
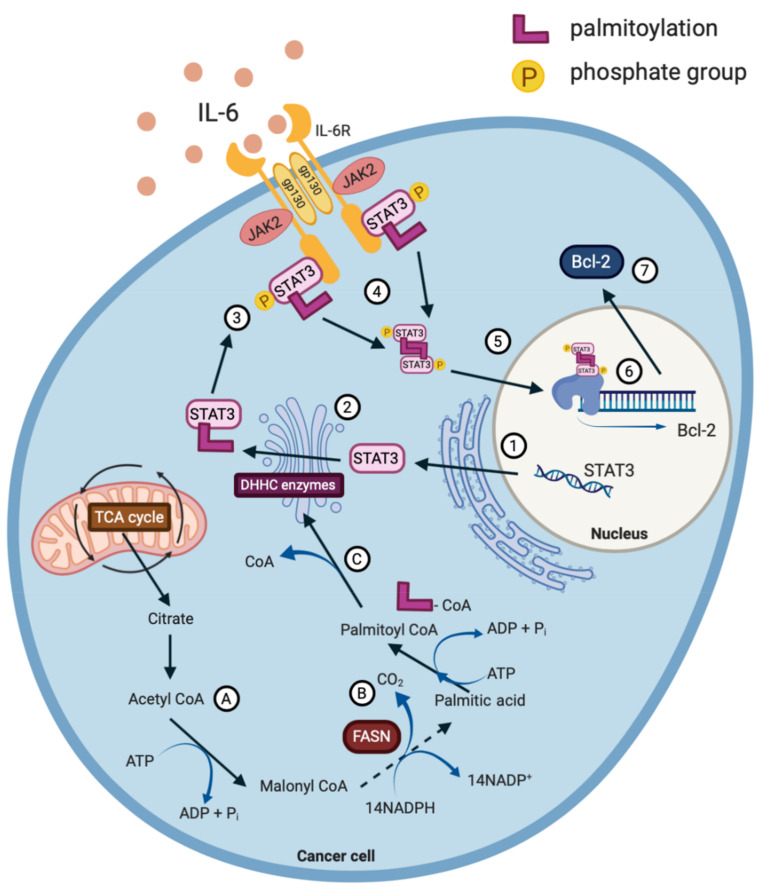
STAT3 is transcribed (1) and subsequently post-translationally palmitoylated by palmitoyl acyltransferase (DHHC) enzymes in the Golgi apparatus (2). IL-6R/JAK/STAT3 signalling is initiated at the plasma membrane by the formation of a hetero-hexametric complex (3), promoting the phosphorylation of STAT3 at Tyr705. Palmitoylated and phosphorylated STAT3 forms a dimer with the aid of the palmitoylation at the SRC homology 2 domain (4) before being translocated to the nucleus (5) and binding to the promoter regions of various oncogenes (6). For example, STAT3 binds to and upregulates the expression of BCL2 (7). The fatty acid moieties required for palmitoylation are synthesized from acetyl CoA (A) from the tricarboxylic acid cycle (TCA); with the help of the enzyme fatty acid synthase (FASN) (B); to yield various long chain fatty acids e.g. palmitic acid. Palmitate is subsequently converted to palmitoyl CoA which can then contribute to the palmitoylation of proteins within the Golgi apparatus (C).

IL-6 signalling initiates the formation of a hetero-hexametric complex that consists of IL-6, IL-6R and glycoprotein 130 (gp130), followed by the recruitment of JAKs and STAT3. The proximity of JAK2 and STAT3 promotes the phosphorylation of STAT3 at tyrosine-705 (Y705) initiating the translocation of STAT3 to the nucleus, where it functions as a transcription factor, initiating the gene expression of the genes involved in tumourigenesis (Figure 2) [38,39]. The phosphorylation of serine-727 (S727) augments the transcriptional effectiveness of STAT3 and potentially permits interaction with other transcription factors, such as nuclear factor kappa B (NF-κB). Furthermore, the increased phosphorylation of S727 correlates with mitochondrial STAT3 metabolic activity in tumours, whereas the prominence of Y705 favours aerobic glycolysis [40]. Once dephosphorylated, STAT3 returns to the cytoplasm in its unphosphorylated form [41]. The importance of the IL-6R/JAK/STAT3 signalling pathway for tumourigenesis is evident, as it is estimated to be aberrantly activated in >70% of human cancers [38,41].

## 2. The Role of STAT3 in Lipid Metabolism

STAT3 is a key regulator of metabolism in cancer progression, orchestrating cellular signalling pathways between the cytosol, nucleus and the mitochondria. The efficacy of inhibiting STAT3 increases under nutrient challenging conditions, suggesting that cancer cells experiencing metabolic stress rely on STAT3 for their survival. As mentioned previously, triacylglycerols are particularly important to cancer cells as an energy source. STAT3 drives the expression of LPL, which catalyses the hydrolysis of triglycerides into free FAs in chronic lymphocytic leukaemia [42]. STAT3 also induces the expression of genes that promote adipocyte lipolysis [43,44]. Carnitine palmitoyltransferase I (CPT1) is important for the transportation of lipids into the mitochondria [45]. Breast cancer cells stimulate the depletion of adipocyte triacylglycerols and transfer free FAs to breast cancer cells, increasing *CPT1A*, resulting in increased proliferation and migration [46]. Mammary adipocytes also secrete leptin, activating the JAK/STAT3 signalling pathway in breast cancer cells, resulting in the upregulation of the *CPT1B* gene and FAO [16]. Furthermore, the adipokine IL-8 promotes the nuclear translocation of STAT3, upregulating the expression of fatty acid binding protein 4 (FABP4), a protein that works synergistically with CD36 to initiate the uptake of FAs [47,48]. FABP4 drives the lipolysis and secretion of FAs from adipocytes and is involved in ovarian cancer cell metastasis to the omentum [14]. Recently, the importance of CD36 became apparent, as the blocking of CD36 with neutralizing antibodies resulted in almost complete inhibition of metastasis in human melanoma, breast and oral cancers, demonstrating cancer cells’ reliance on dietary lipids to promote metastasis [49].

### 2.1. JAK/STAT3 Pathway as a Therapeutic Target

As discussed, the transcription factor STAT3 is associated with tumourigenesis in several types of cancers. Thus, STAT3 represents an attractive therapeutic target. However, the focus of drug design has relied on targeting the STAT3 canonical pathway, involving STAT3 phosphorylation (Y705), its dimerization and translocation to the nucleus. This approach has been highly challenging as the majority of these small molecule inhibitors interfere with the binding of the SRC homology 2 (SH2) domain, but STAT3 transcriptional activity is often not completely inhibited [38]. More recently, the focus has been on the targeted degradation of STAT3. For instance, Bai et al. recently demonstrated the efficacy of a proteolysis targeting chimaera (PROTAC) for STAT3. This PROTAC demonstrated complete tumour regression in mouse models of blood cancers [50].

Ruxolitinib is a small-molecule inhibitor of JAK1/2 proteins, both of which have been detected in adipocytes [44]. By inhibiting JAK2 proteins, it is possible to indirectly interfere with the phosphorylation of STAT3, rendering it inactive. Ruxolitinib is currently used in ongoing clinical trials in patients with breast, colorectal, head-and-neck, lung, ovarian, pancreatic, and prostate cancers, following its success in murine models of ovarian cancer [38]. Moreover, various monoclonal antibodies targeting IL-6 signalling, such as Situximab and Tocilizumab, have demonstrated antitumour efficacy against cancer, further solidifying the importance of IL-6 in tumour growth [38].

### 2.2. Post-Translational Modification of STAT3 as a Therapeutic Target

Post-translational palmitoylation was recognized ~25 years ago and is carried out by a family of transmembrane proteins known as palmitoyl acyltransferases. The common abbreviation DHHC refers to the Asp-His-His-Cys motif within the enzymes structure [51]. This DHHC family of enzymes attaches FA residues to cysteine-rich sites within proteins of interest to aid cellular localization [52,53]. Genetic alterations to palmitoyl acyltransferases have been observed in a variety of diseases with the high expression of these enzymes usually being a marker for poor prognosis in cancer (excluding ZDHHC14 that has now been identified as a tumour suppressor gene) [54,55,56].

Recent studies have begun to focus on the post-translational modification of STAT3 as a therapeutic target for cancer treatment. By targeting this modification and altering the chemical structure of STAT3, it is possible to impair the function of the transcription factor and reverse its oncogenic effects. For example, STAT3 is post translationally S-palmitoylated [57] (Figure 2), much like other oncoproteins such as R-Ras; a protein highly associated with malignant transformation [58]. It is the constitutive cycles of de/re-palmitoylation that orchestrate the cellular distribution of Ras proteins [59].

The mechanism of palmitoylation can be broken down into two main steps; first, the palmitoylation of the palmitoyl-acyltransferase enzyme, followed by the transfer of the palmitoyl group from the enzyme to the target protein [60]. Additionally, it is possible for some proteins to undergo auto-palmitoylation, a process independent of palmitoyl acyltransferase enzymes [61]. However, at physiological concentrations of lipid, the rate of spontaneous palmitoylation is expected to be too slow to contribute significantly to the total palmitoylation of signalling proteins in mammalian cells. Therefore, palmitoyl acyltransferase enzymes are likely to represent the predominant mechanism of palmitoylation in vivo [62,63]. In the context of dysregulated lipid metabolism-associated cancers, physiological concentrations of lipids may be increased, meaning that the rate of auto-palmitoylation may also be higher.

## 3. The Role of STAT3 and Lipid Metabolism in Therapy Resistance

Obesity is known to have a role in cancer development and therapy resistance, but the mechanisms remain largely undefined. Programmed death-1/Programmed death ligand 1 and 2 (PD-L1/2) and Cytotoxic T-lymphocyte associated protein 4 (CTLA-4) are immune checkpoint proteins involved in antitumour immune responses. PD-L1 or 2 expression by tumours mediates the robust inhibitory signals to T effector cells, impacting antitumour immunity. Extensive studies show significant improvement in cancer immunotherapy with antibodies targeting CTLA-4 (approved antibody ipilimumab) and PD-1/PD-L1 in various cancer types [64,65,66,67]. Recently, it has been shown that an obesity-associated increase in FAO in CD8^+^ T effector cells was orchestrated by the Programmed death-1 (PD-1) activation of STAT3 signalling, and this pathway had a key role in breast cancer progression. Furthermore, the adipokine leptin abrogated CD8^+^ T effector cell function via the STAT3- and FAO-mediated inhibition of glycolysis [68]. Upon activation, T cells reroute their metabolic programming to increase the rate of FAO. PD-1 has been shown to promote the FAO of endogenous lipids via the increased expression of *CPT1A* [69]. STAT3 silencing in breast cancer cells or the ablation of T cell *STAT3* led to a reduced STAT3-mediated PD-L1 expression, and reduced breast tumour development [65,68].

STAT3 and the accompanying signalling pathways have been associated with multiple mechanisms of resistance to cancer therapies [70]. Chemotherapy resistance is one of the major reasons for the poor survival rates in many cancers. Relatively high levels of phosphorylated STAT3 are known to correlate with the degree of cisplatin resistance in ovarian cancer models [71,72]. Chemotherapy resistance in breast and bladder cancer is orchestrated by JAK/SAT3-regulated CPT1B expression and FAO [16,73]. Leukemic stem cells evade chemotherapy by occupying bone marrow adipose tissue niche. Adipocytes promote acute myeloid leukaemia cell survival via FAO. Furthermore, recent studies identified FAO as a driver of drug resistance in acute myeloid leukaemia. The adipokine adiponectin, released from bone marrow adipocytes, was demonstrated to have a role in chemotherapy resistance in myeloma cells [18,74,75]. Whereas leptin, an adipokine with a central role in obesity, induced JAK2/STAT3 signalling, leading to the promotion of breast cancer stem cell (CSC) survival and self-renewal [76]. Previous studies have shown that inhibiting JAK/STAT3 signalling in CSCs resulted in reduced tumour growth in vivo and interfered with CSC self-renewal [16,77,78]. Adipocytes upregulate CD36 and drive cancer cells to gain CSC-like features [79]. Adipocyte-derived factors induce IL-6 production by cancer cells protecting cancer cells from radiation therapy [80]. The stimulation of cancer cells with IL-6 was shown to abrogate sensitivity to cisplatin and increased the formation of tumour spheres induced by high-fat diets in in vivo mouse models of lung cancer [71]. Therefore, due to widespread therapy resistance in cancer, there is a need for alternative or complimentary strategies for cancer prevention and treatment.

## 4. Phytochemicals

Natural products play a large role in the discovery and development of drugs for the treatment of cancer and the prevention of drug resistance [81,82]. Phytochemicals are able to target various stages of tumourigenesis and are considered safer and better tolerated with lower toxicity compared to current chemotherapeutic drugs. However, phytochemicals show a lack of target specificity and due to the differences between in vitro experiments and human physiological conditions, the antitumour effects of phytochemicals in the laboratory have, on the whole, not been successfully translated to a clinical setting. Although research is undergoing to improve the formulations of phytochemical compounds for better therapeutic outcomes [83], the use of phytochemicals as natural inhibitors of STAT3 has shown promise in various cancer cell lines and models (Table 1).

### 4.1. Apigenin

Apigenin is a flavonoid found in fruits and vegetables such as parsley, onions and oranges. The anticarcinogenic properties of apigenin arise via the suppression of inflammation, angiogenesis, cell proliferation and the induction of autophagy and apoptosis [93]. Apigenin has also decreased cancer cell motility, and cancer cell migration and invasion, by modulating multiple pathways including JAK/STAT [94].

As discussed previously, STAT3 is often highly active in drug-resistant cancers [95]. By decreasing STAT3 signalling, apigenin reversed drug resistance by suppressing drug efflux in adriamycin-resistant MCF-7 breast cancer cell lines. Apigenin reduced the expression of *MDR1*, multidrug resistance-associated proteins (*MRP1*, *MRP3*, *MRP5*) and breast cancer-resistant proteins (BCRP) [96]. In addition to its role in inhibiting drug resistance, apigenin reduced cell migration and invasion in the melanoma A375 cell line by decreasing STAT3 phosphorylation, nuclear localization and transcriptional activity, in addition to STAT3 target genes, *MMP2*, *MMP9*, *VEGF* and *TWIST1* [84].

The anti-inflammatory properties of apigenin were observed in inflammatory bowel disease and colitis-associated cancer. Furthermore, apigenin reduced colonic damage and reduced the severity of colitis mouse models of inflammatory bowel disease, decreasing cytokine levels (TNFα, IL-1β, IL-6, MCP-1 and CSF-1) in a concentration-dependent manner. Apigenin was demonstrated to inhibit inflammation and inflammation-induced carcinogenesis by suppressing STAT3 and NF-κB signalling in vivo, and showed a protective effect against colitis-associated cancer by reducing the tumour volume in addition to decreasing neutrophil infiltration [97].

Apigenin has also been shown to inhibit adipogenesis by targeting STAT3/CD36 signalling. The reduction of STAT3 phosphorylation reduced the expression of the *CD36* and STAT3 target genes, *AP2* and *SCD*. As a result, lower levels of free FAs and lipid accumulation were observed in adipocytes treated with apigenin [98,99].

### 4.2. Cucurbitacin B and I

Cucurbitacins are triterpenes, isolated from the Cucurbitaceae family of plants that include cucumbers, pumpkins and gourds. Cucurbitacins display anti-inflammatory, cytotoxic and antitumour properties [85,100]. Cucurbitacin B and I exhibit antiproliferative effects on several cancers, including glioblastoma [101], breast [102], lung [86,100] and osteosarcoma [103].

Cucurbitacin B results in G_2_/M phase arrest and the induction of the caspase cascade as a result of STAT3 inhibition in cancer cells [85,101,104,105]. In pancreatic cancer cells, JAK2/STAT3 signalling, as well as STAT5, were inhibited by cucurbitacin B, which resulted in the decreased expression of cyclin A, cyclin B1 and Bcl-xL, leading to the induction of apoptosis. Cucurbitacin B treatment showed antitumour activity and potentiated the anti-proliferative effects of chemotherapeutic drugs gemcitabine and Adriamycin in in vivo models of pancreatic carcinoma and myeloma, respectively [85,105]. In myeloma cells, cucurbitacin B exhibits antitumour properties by inhibiting IL-10 induced STAT3 phosphorylation, suppressing proliferation and promoting apoptosis via p38 activation [105].

Cucurbitacin I suppressed STAT3 phosphorylation, downregulating STAT3 targets Mcl-1, c-Myc, cyclin D1 and survivin and upregulating the cleavage of PARP proteins in vitro and in vivo models of osteosarcoma. In osteosarcoma cells, cucurbitacin I reduced the cell viability and induced apoptosis. Mice models of osteosarcoma receiving cucurbitacin I treatments showed improved survival, body weight and tumour cell apoptosis, resulting in the suppression of tumour growth [103]. The pro-apoptotic effect of cucurbitacin I was seen in B-leukaemia cells and primary chronic lymphocytic leukaemia, where the inhibition of phosphorylated STAT3 led to the downregulation of an anti-apoptotic gene, *XIAP*, and the cell cycle regulatory gene, *CDC2* and upregulated *DR4* [106]. Cucurbitacin I was also able to induce pro-death autophagy signalling in lung carcinoma cells through ERK/mTOR/STAT3 signalling by inhibiting ERK activation and the downstream phosphorylation of mTOR and STAT3 [86]. Additionally, the treatment of lung carcinoma cells with cucurbitacin I inhibited IL-6 induced STAT3/JAK2 signalling [100].

Activated STAT3 stimulates the regulation of genes encoding adipogenesis markers PPARγ, aP2, C/EBPα, adiponectin and CD36. Both cucurbitacin B and I exhibited anti-adipogenesis activity by suppressing adipocyte differentiation and lipid accumulation as a result of STAT3 inhibition [107].

### 4.3. Curcumin

Curcumin is the major bioactive polyphenol found in turmeric, the ground rhizome of *Curcuma longa*. Curcumin displays antioxidant, antitumour, anti-angiogenesis, and chemotherapeutic properties [108].

Curcumin has been widely studied for its role as an anti-obesity agent by mediating adiposity and lipid metabolism [108]. Curcumin targets lipid metabolism by inhibiting adipocyte differentiation and fat accumulation by suppressing fatty acid synthase (FAS) and increasing FAO [109]. A lower expression of *Pparg* and *Cebpa* was observed in the subcutaneous adipose tissues which may be associated with reduced body weight gain and adiposity in high-fat diet fed mice [110].

The antitumour properties of curcumin have been observed in cancers such as ovarian [111], breast [112], bladder [113], osteosarcoma [114], retinoblastoma [87] and oesophageal [115], which may be partly due to its ability to prevent inflammation by suppressing proinflammatory transcription factors, such as NF-κB [113,115], which leads to the downregulation of adipokines such as IL-6 [111] and leptin [109], and the upregulation of adiponectin, which affects lipid homeostasis [109,116]. In ovarian cancer cells, curcumin inhibited the lysophosphatidic acid-induced secretion of IL-6 and IL-8 by inhibiting STAT3 phosphorylation, resulting in reduced cancer cell motility [111].

In several cancers, curcumin-targeted JAK/STAT signalling caused a reduction in cell proliferation and migration and the induction of apoptosis [87,114]. In osteosarcoma cells, curcumin was able to reduce growth viability and survival in addition to blocking the cell cycle progression of the G_2_/M phase as a result of JAK2/STAT3 inhibition [114]. Curcumin also inhibited viability, migration and invasion and induced apoptosis in retinoblastoma cells through the suppression of JAK1, STAT1 and STAT3 phosphorylation. The reduced migratory and invasive capabilities as a result of curcumin treatment were due to a decrease in the expression of *MMP2*, RHOA, ROCK1 and Vimentin [87]. In addition to inducing apoptosis, curcumin could potentiate the apoptotic effects of chemotherapeutic agents, paclitaxel and gemcitabine in bladder cancer cells [113].

### 4.4. Epigallocatechin Gallate (EGCG)

Green tea has been well researched and is recognized to contribute to many health benefits due to the high concentrations of polyphenols green tea contains. Epigallocatechin gallate (EGCG) comprises 40% of the total phenolic mixture of green tea catechins. Studies have suggested that green tea possesses antioxidant, anti-inflammatory and antitumour properties [108].

Evidence indicates that EGCG can prevent STAT3 activation by binding and interacting with the Arg-609 residue within the STAT3 SH2 domain, the domain responsible for STAT3 and peptide binding [88]. The inhibition of STAT3 by EGCG may be responsible for its antitumour and anti-adipogenic effect [88,117,118,119,120]. In hepatocellular carcinoma, the EGCG-mediated inhibition of STAT3 phosphorylation led to the reduction of downstream target genes encoding Bcl-xL, c-Myc, VEGF and Cyclin D1, resulting in the suppression of cell proliferation and the induction of apoptosis [88]. The adipokine IL-6 is able to promote tumour growth and metastasis, while EGCG was able to inhibit IL-6 induced *VEGF* expression and angiogenesis in both in vitro and in vivo models of gastric cancer by suppressing STAT3 activity [121]. EGCG-mono-palmitate (EGCG-MP), the most stable derivative of EGCG, induced SHP-1, which led to a decreased expression of the oncogenic protein BCR-ABL and a reduction in STAT3 phosphorylation, resulting in cytotoxicity and the induction of apoptosis in chronic myeloid leukaemia [117].

EGCG has been widely studied for its role as an anti-obesity agent, due to its ability to suppress adipocyte differentiation and proliferation [108]. EGCG demonstrated a suppressive action on lipid accumulation and pro-inflammatory cytokines in vitro and in vivo [120,122,123]. Palmitic acid (PA) has been shown to increase the invasiveness of several cancers via the induction of lipid accumulation and proinflammatory cytokine release [118,123]. The treatment of PA-stimulated microglia cells with EGCG prevented JAK2 and STAT3 phosphorylation, and attenuated lipid accumulation and inflammatory responses [123]. High-fat diet-fed mice receiving EGCG supplements showed a reduction in bodyweight, lipid deposition and adipocyte size, in addition to a decline in inflammatory cytokines such as TNFα, IL-6 and IL-1β, via the suppression of NF-κB [120,123]. EGCG can block adipocyte differentiation via reducing the expression of VEGF, C/EBPα and PPARγ [118,119].

### 4.5. Resveratrol

Resveratrol is a stilbenoid polyphenol, abundant in grape skins, peanuts and red wine. Resveratrol has been implicated in preventing the development of disorders such as cardiovascular disease, diabetes, cancer and obesity. The beneficial effect of resveratrol on obesity may be attributed to its ability to increase AMPK phosphorylation and activation, resulting in the upregulation in FAO [108]. The targeting of STAT3 signalling by resveratrol have demonstrated anti-cancer and antitumour activity in many cancers [89,90,124,125,126,127,128,129].

In breast cancer, resveratrol treatment was able to inhibit proliferation, migration and invasion when treated with cancer-associated fibroblast-conditioned media. Resveratrol reduced the expression of genes encoding Cyclin D1, c-Myc, *MMP2*, *MMP9* and the protein expression of SOX2 as well as Akt and STAT3 activation, which may reduce self-renewal and stemness [90]. Lipogenesis plays a role in self-renewal and proliferation. In a breast cancer model, lipogenic genes, *ACLY*, *ACC1*, *SREBP1* and *NFASN* were upregulated. Treatment with resveratrol led to a reduction in lipogenesis, accompanied by a significant downregulation of lipogenic genes, including *NFASN* expression, and upregulated the pro-apoptotic gene *DAPK* and *BNIP3* leading to tumour cell death and apoptosis [125,126].

Resveratrol was an effective treatment in constitutively active STAT3 cancer cells. The treatment of malignant cells with resveratrol displayed irreversible cell cycle arrest in breast, pancreatic and prostate carcinoma, resulting in a loss of viability and the induction of apoptosis. STAT3 targets Cyclin D1, Bcl-xL and Mcl-1 were also repressed by resveratrol treatment [124]. The antitumour effect of resveratrol can be seen in lung cancer cells that have been co-cultured with human macrophages. Resveratrol inhibited lung cancer progression by the pro-tumour activation of tumour-associated macrophages, in addition to decreasing the tumour growth associated with cell proliferation suppression [127]. Triacetylresveratrol (TRES) is the acetylated analogue of resveratrol displaying improved pharmacokinetic properties, with a longer half-life and increased volume of distribution. The hydrophobic nature of TRES facilitates increased interaction with phospholipid bilayers in comparison to resveratrol. Both TRES and resveratrol suppressed pancreatic cell growth and induced apoptosis via the inhibition of STAT3 and NF-κB activation, downregulating Mcl-1 and the upregulation of the proapoptotic proteins BIM and PUMA. [89].

### 4.6. Silibinin

Silibinin is the bioactive polyphenolic flavonoid isolated from the seeds of the herb milk *thistle* (*Silybum marianum*). Silibinin exhibits promising antitumour effects by targeting multiple cell signalling pathways in vivo and in vitro. Studies have shown that silibinin can reduce therapy-associated nephrotoxicity, neurotoxicity and cardiotoxicity in preclinical models, and carries the potential to reverse cancer cell drug resistance [130,131]. Silibinin is a natural STAT3 inhibitor which may be responsible for its antitumour properties in many carcinomas, such as prostate, endometrial and lung [91,92,131,132,133,134,135,136,137,138].

Silibinin appears to be effective against tumours by inhibiting viability and cell proliferation, decreasing the amount and size of tumours and promoting apoptosis [133,135,136,137]. The antitumour effect of silibinin may correlate with anti-angiogenic mechanisms mediated by the upregulation of angiogenic inhibitors, ANG-2 and TIE-2, and decreasing the production of tumour-associated macrophages and cytokines that are associated with the inhibition of NF-κB and STAT3 phosphorylation [136]. In prostate carcinoma, silibinin reduced the expression of VEGF which may be responsible for its anti-angiogenic activity. Silibinin could also target cell cycle progression by downregulating the expression of JNK1/2, CDKs and cyclins and upregulating ERK1/2 and p38 MAPK phosphorylation to inhibit cell proliferation and promote apoptosis [134].

The expression of the STAT3 target gene, c-Myc was reduced following silibinin treatment which induced metabolic reprogramming [133,138]. In pancreatic carcinoma, silibinin appeared to impair glycolysis that resulted in the reduced utilization of the pentose phosphate pathway and nucleoside synthesis. The inhibition of *GLUT1* and *HK2* expression by silibinin may be responsible for the reduced glucose uptake and glycolytic activity inhibiting cancer cell growth and proliferation [133]. Metabolic reprogramming may contribute to the antitumour effect of silibinin. In endometrial cells, silibinin also has a lipid homeostatic role where treatment with silibinin can prevent STAT3 activation and suppress *SREBP1* expression leading to a reduction in lipid accumulation [91].

### 4.7. Palmitoylation and Phytochemicals

The purpose of palmitoylation is to serve as both a way of tethering proteins to membranes and directing their localization to membrane microdomains by increasing their hydrophobicity [61,139]. Some phytochemicals have exhibited the ability to ‘block’ palmitoylation by specific DHHC enzymes that consequentially results in impaired function. For example, curcumin blocks the auto-palmitoylation of DHHC3, the enzyme responsible for the palmitoylation of integrin β4, a signalling molecule upregulated in breast cancer cells. Treatment with curcumin prevented the palmitoylation of integrin β4 that resulted in the decreased invasiveness of breast cancer cells [140]. These results suggest a promising future for phytochemicals as cancer therapies targeting DHHCs, when it is taken into consideration that the high expression of DHHC enzymes is a negative prognostic marker in several cancers [55]. In contrast, resveratrol restored the deleterious effect on CPT-1 in diet-induced obese mice. As a consequence, cell-mediated immunity was improved [141]. While this was particularly advantageous for preventing infectious disease, in the context of cancer, this increase in CPT-1 would perhaps not be so advantageous, considering that CPT-1 has been shown to aid cancer progression by contributing to cancer cell senescence [142].

STAT3 is palmitoylated in the endoplasmic reticulum initiating a signalling cascade that is depicted in Figure 2 [55]. Considering that the IL-6R/JAK/STAT3 pathway is aberrantly hyperactivated in >70% of cancers, it would be interesting to determine if impairing the palmitoylation of STAT3 with the use of phytochemicals would prove to be successful in hampering cancer cell growth [38]. When reviewing the literature, it appears that the targeting of specific palmitoyl acyltransferases with phytochemicals is yet to be carried out in the context of cancer. Therefore, this is an intriguing future prospect for targeting STAT3 as a mediator of cancer progression. In addition, it has been shown that a number of DHHC enzymes can be deleted in mice without causing any major defect to animal development, further validating the targeting of the DHHC enzymes with phytochemicals as a promising cancer therapy [55].

Another potential route of exploring post-translational modification as a target for phytochemicals is via epigenetics. In fact, STAT3 is itself known to contribute to epigenetic silencing via the DNA methylation of tumour suppressor genes. Identifying a phytochemical with the ability to reverse this hypermethylation would be a beneficial cancer treatment [143]. Epigenetic alterations involve similar secondary modification as described above, but rather than the palmitoylation of a protein, epigenetics refers to the methylation, acetylation or deacetylation of DNA and is essential for the regulation of gene expression [144,145]. Dietary phytochemicals have demonstrated the influence on the human epigenome which comes as no surprise when it is considered that epigenetic modifications are known to be reversible. Polyphenols, such as EGCG from green tea, have demonstrated efficacy in inhibiting DNA methyltransferases, and their ability to silence tumour suppressor genes in models of prostate cancer [146]. EGCG decreased levels of DNA methylation in triple-negative breast cancer cells, limiting the migration of the breast cancer cells as a consequence [147]. In addition, curcumin reversed the alterations in DNA CpG methylation of Tnf in a mouse model of colon cancer [148].

Research into the targeting of secondary modifications with phytochemicals seems to have been relatively inconsistent over the past two decades. The reason for this may be that phytochemicals are themselves palmitoylated, making them a contradictory choice of therapy when it comes to targeting palmitoylation. However, studies such as those by Coleman et al. have shown that by focusing on a minor part of a complex chemical structure it is possible to have an advantageous deleterious effect in the context of cancer. There are still many gaps in knowledge when it comes to the specific palmitoylation of STAT3, reiterating the novelty and specificity of this field. Although, farnesyl transferase inhibitors have now been assessed in clinical trial, so it is likely that palmitoyl acyltransferase inhibitors will begin to receive similar attention in the coming years [149].

### 4.8. Phytochemicals and Immunotherapy

**Figure 3:**
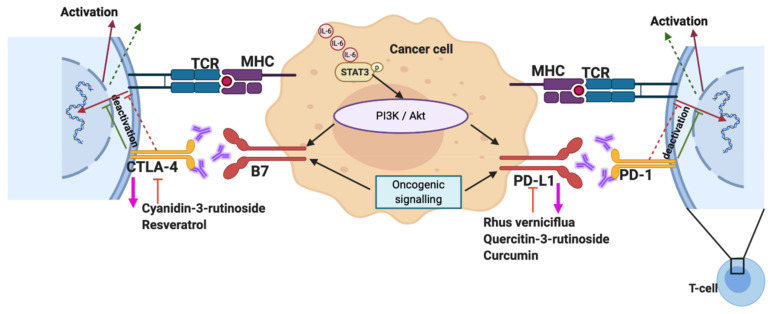
Mechanism of the phytochemicals targeting the immune checkpoint receptors. T-cell receptor: TCR; major histocompatibility complex: MHC; cytotoxic T-lymphocyte-associated protein 4: CTLA-4; programmed death ligand 1: PDL-1.

Phytochemicals in their naturally active form have also shown promise as immunomodulatory agents in cancer. For example, the phytochemical shikonin, isolated from the root tissues of the herb *Lithospermum erythrorhizon*, has been demonstrated to enhance the immunogenic cell death response toward tumour cells, augmenting dendritic cell vaccine activity in vivo, increasing cytotoxic T cell activity and reducing tumour growth, often via the release of damage-associated molecular patterns (DAMPs) [150,151,152]. In addition, pretreating cancer cells with shikonin results in a reduced effective dose of the chemotherapy agent doxorubicin, giving promise to the strategy of using phytochemicals in the development of dendritic cell vaccines and in the use of combinatorial drug treatments [153]. Shikonin has been demonstrated to target the SH2 domain of STAT3 resulting in the suppression of IL-17-induced vascular endothelial growth factor (VEGF) expression, sensitization to gefitinib and the induction of tumour cell apoptosis [154,155,156,157]. Other phytochemicals, such as *Ganoderma lucidum* polysaccharides, have been demonstrated to alter the ratio of lung tumour associated CD4^+^ and CD8^+^ T cells [158]. Whilst curcumin has also shown promise in restoring progenitor, effector and circulating T cells in tumour-bearing mice, via Stat5a-mediated Bcl-2 induction [159]. Recent studies have also shown the beneficial therapeutic effect of phytochemicals on STAT3-mediated PD-1 and CTLA-4 expression on T cells (Figure 3). The active compounds in black raspberry extract (Cyanidin-3-Rutinoside, Quercitin-3-rutinoside) reduced the expression of immune checkpoint receptors CTLA-4 and PD-1 and affected CD4^+^ and CD8^+^ T lymphocyte proliferation, whilst reducing the in vitro expansion of myeloid-derived suppressor cells [160]. In a high-fat diet-induced obesity mouse model, resveratrol significantly reduced CTLA-4 expression in regulatory T cells [161], demonstrating results similar to those reported elsewhere in an inflammatory bowel disease model [162,163]. Furthermore, studies show that active compounds in *Rhus verniciflua* Stokes extract, used in Korean herbal medicine, successfully blocked the expression of both PD-1/PDL-1 and CTLA-4 [164,165,166].

## 5. Conclusions

STAT3 signalling, across several cell types in the tumour microenvironment, plays a major role in tumour development and progression. This is due to STAT3 playing a key role in the regulation of multiple metabolic pathways that contribute to tumourigenesis, including lipid metabolism. Phytochemicals demonstrate a diverse array of antitumour properties by targeting STAT3 directly or downstream targets as well as the STAT3 orchestration of lipid metabolism. Apart from cucurbitacins, the phytochemicals discussed in this review are natural, low cost and display low toxicity, making them potential candidates as STAT3 inhibitors in chemoprevention. Phytochemicals may also be used in combination with other therapeutic drugs in order to enhance the therapeutic potential or resensitize chemotherapy-resistant cancers to therapeutic drugs. However, the clinical use of phytochemicals as an anticancer therapy is limited due to poor bioavailability and stability, in addition to inadequate knowledge about the mechanism of action and potential offset hazards. As STAT3 signalling is essential for cell functions, including immune regulation, the pleiotropic effect of inhibiting STAT3 may raise the risk of adverse reactions downstream. Future work should be carried out to address these challenges before phytochemicals can be used as or in complementation to antitumour therapeutic agents. 

## Figures and Tables

**Table 1:** Effects of the phytochemicals on STAT3 signalling and the targets in different cancer cell line models.

Phytochemical	Cell Line/Model	Molecular Mechanism	Effect	Ref
**Apigenin**	A375	↓pSTAT3 ↓*MMP2* ↓*MMP9* ↓VEGF ↓TWIST1 ↓E-cad ↓Keratin-8 ↑N-cad ↑Fibronectin	- Inhibition of cell migration and invasion - Partial reversal of epithelial mesenchymal transition (EMT)	[84]
**Cucurbitacin B**	Panc-1	↓pSTAT3 ↓pSTAT5 ↓pJAK2 ↓Cyclin A ↓Cyclin B1 ↓Bcl-xL ↑Caspase-3 ↑Caspase-9	- Inhibition of proliferation - Induction of G_2_/M cell cycle arrest and apoptosis	[85]
**Cucurbitacin I**	A549	↓pSTAT3 ↓p-mTOR ↓pERK ↑LC3II	- Decrease in cell viability - Inhibition of colony formation - Induction of autophagy	[86]
**Curcumin**	SO-Rb50, Y79	↑*MIR99a* ↓pJAK1 ↓pSTAT1 ↓pSTAT3 ↓*MMP2* ↓RHOA ↓ROCK1 ↓Bcl-2 ↓Vimentin ↑Bax	- Inhibition of migration and invasion - Induction of apoptosis	[87]
Epigallocatechin Gallate (**EGCG**)	BEL-7402, OGY-7703	↓pSTAT3 ↓Bcl-xL ↓c-Myc ↓VEGF ↓Cyclin D1	- Suppression of cell proliferation - Induction of apoptosis	[88]
**Resveratrol**	PANC-1, BxPC-3	↓pSTAT3 ↓pNF-κB ↓Mcl-1 ↑BIM ↑PUMA	- Inhibition of cell viability - Induction of apoptosis	[89]
	MCF7, MDA-MB-231	↓pSTAT3 ↓pAkt ↓*MYC* ↓*MMP2* ↓*MMP9* ↓*SOX2* ↓*BMI1* ↓*CD44*	- Inhibition of cancer associated fibroblast-induced migration, invasion and self-renewal	[90]
**Silibinin**	Ishikawa, RL-952	↓pSTAT3 ↓*SREBP1* ↓FAS ↓p*ACLY* ↓Survivin ↓Bcl-2 ↓Caspase-3 ↓Ki67 ↓Cyclin D1	- Reduced lipid accumulation - Inhibition of proliferation and cell viability	[91]
	DU145	↓pSTAT3 ↓Mcl-1 ↓Cyclin D1 ↓Bcl-xL ↓Survivin	- Induction of apoptosis	[92]
